# On the Normal Degree of the Temperature of Man

**Published:** 1852-11

**Authors:** E. Brown Séquard

**Affiliations:** Paris


*On the Normal Degree of the Temperature of Man.* By E. Brown Séquard, M. D., of Paris. The degree of animal heat, in the human species, is stated as being between 98.5° and 100° F., (37 and 38° Cs.) I intend to prove that it is higher.

It is impossible to take directly the temperature of most of the internal parts of the body in man; therefore many physiologists, in order to discover the temperature of these parts, have argued as follows: According to J. Hunter, the temperature of the rectum in animals is the same as that of the right ventricle of the heart; hence it has been concluded that as the temperature of the rectum, in man—still according to Hunter—is 98°.42 F., (36°.9 Cs., the temperature of the internal parts of the body ought to be between 98 and 99° F., (36°.67 and 37°.22 Cs.)

Some other physiologists, noting the temperature of the mouth under the tongue, and supposing that this temperature is nearly the same as that of the internal parts of the body, have concluded that the temperature of man is between 99 and 100° F., (37°.2 and 37°.8 Cs.)

We will prove that these deductions are not right:

Firstly, the degree of the temperature of the rectum is not, as Hunter says, 98°.4. It may be so in debilitated men, but in healthy persons it is more elevated. It is between 100 and 102° F., (37.7 and 38°.89 Cs., according to my own researches and to those of Berger and Maunoir. Besides, many experiments on dogs, rabbits and Guinea pigs, have shown satisfactorily to myself that the temperature of the rectum is not equal to that of the right ventricle. This last organ is from 1 to 3° F., (0.55 to 1.7° Cs.,) higher than the rectum.

The temperature of the rectum, in man, being between 100 and 102° F., (37°.7 and 38°.8 Cs.,) and the temperature of the right ventricle, in man, being from 1 to 30° F., (0.56 to 1.7 Cs.,) higher than that of the rectum, if we suppose the same difference existing in man as in mammals, it follows that the temperature of the right ventricle of the heart in man ought to be between 101 and 105° F., (38°.33 and 40.56 Cs.)

But a closer approximation of the exact temperature of the internal parts of the body, in man, may be obtained by taking the temperature of an organ situated deeper than the rectum, and, consequently, less exposed to the influence of the atmosphere. Such is the case in the bladder. I have observed its temperature by taking that of the urine at the moment of its emission, and before any sensible change had occurred in it. The urine was directly received in a vase dipped into a large quantity of water at 98° F., (36°.7 Cs.) Thus I have ascertained that the mean temperature of the urine, in man, is 102°.6 F., (39°.2 Cs.)

Now if we take notice of this well known fact, that the temperature of the lower part of the abdomen is less elevated than the upper part, we are authorized to believe that the temperature of the central parts of the body, in man, is very near 103° F., (39°.5 Cs.)

My experiments on the temperature of urine were made on ten strong sailors, in the spring, on the Atlantic Ocean, between the 43d and 45th deg. of north latitude. The lowest degree of the temperature of urine which I have observed, was 100°.9 F., (38°.3 Cs.;) the highest was 103°.2 F., (39°.56 Cs.) My own urine, examined more than thirty times, in the most varied conditions, has been nearly always at the same temperature; the variations have been only between 102° and 102°.8 F., (38°.89 and 39°33 Cs.) The ordinary degree is 102°.5 F., (39°.17 Cs.)

Long before my researches, the temperature of urine, in the human species, had been taken by some observers, but in general without sufficient care. The temperature of the urine is 94°.25, according to Braun; 98°.9, according to De Lisle; and 103°, according to Hales. Recently

Berger has taken the temperature of urine in the bladder in five women. He has found it equal to 101°.48 F., (38°.6 Cs.)

As the temperature of woman is a little inferior to that of man, the result obtained by Berger is in accordance with mine.

If we take the average between the temperature of the urine of man as I have found it, and that of woman as Berger has found it, we have a number very near 102° F., (389 Cs.)

From these facts we draw the conclusion that the temperature of the thoracic and abdominal viscera, in the human species and in both sexes, is between 102 and 103° F., (38°.89 and 39°.44 Cs.,) *i. e.*, some degrees higher than it is generally admitted.—*Ib.*

